# Transporting observational study results to a target population of interest using inverse odds of participation weighting

**DOI:** 10.1371/journal.pone.0278842

**Published:** 2022-12-15

**Authors:** Albee Y. Ling, Rana Jreich, Maria E. Montez-Rath, Zhaoling Meng, Kris Kapphahn, Karen J. Chandross, Manisha Desai

**Affiliations:** 1 Division of Biomedical Informatics Research, Department of Medicine, Quantitative Sciences Unit, Stanford University School of Medicine, Palo Alto, CA, United States of America; 2 Sanofi, Bridgewater, NJ, United States of America; 3 Division of Nephrology, Department of Medicine, Stanford University School of Medicine, Palo Alto, CA, United States of America; Qatar University, QATAR

## Abstract

Inverse odds of participation weighting (IOPW) has been proposed to *transport* clinical trial findings to target populations of interest when the distribution of treatment effect modifiers differs between trial and target populations. We set out to apply IOPW to *transport* results from an observational study to a target population of interest. We demonstrated the feasibility of this idea with a real-world example using a nationwide electronic health record derived de-identified database from Flatiron Health. First, we conducted an observational study that carefully adjusted for confounding to estimate the treatment effect of fulvestrant plus palbociclib relative to letrozole plus palbociclib as a second-line therapy among estrogen receptor (ER)-positive, human epidermal growth factor receptor (HER2)-negative metastatic breast cancer patients. Second, we *transported* these findings to the broader cohort of patients who were eligible for a first-line therapy. The interpretation of the findings and validity of such studies, however, rely on the extent that causal inference assumptions are met.

## 1. Introduction

Real-world evidence (RWE) is playing an increasingly important role in clinical decision making, especially when clinical trial data are not available or when trial samples do not represent target populations of interest [1,2]. Compared to clinical trials, real-world data (RWD) is less costly to collect, can come from a variety of sources, and usually has larger size [3]. Despite their shortcomings such as data quality issues, RWD can be used for a variety of tasks such as target-drug combination discovery, drug-repurposing, or pragmatic trials [3,4]. The primary way of generating RWE from RWD is observational studies [5–9]. Such studies complement clinical trials in providing evidence for medical practice and regulatory approval of drugs and devices [1,2,4,10].

Propensity score (PS) methods have been developed to address various sources of bias in observational studies. Specifically, inverse probability of treatment weighting (IPW) is one of the fundamental methods to address confounding bias resulted from nonrandom treatment assignment [11]. It can also be used to handle missing data [12]. More recently, IPW has emerged as another popular means to standardize clinical trial results, i.e. to correct for the bias of non-representativeness of trial population compared to target population [5,13–18]. When the trial is a subset of target population, this class of studies are referred to as *generalizability* studies and inverse probability of participation weighting is applied. In case when target population does not overlap with the original trial study population, inverse odds of participation weighting (IOPW) can be used for standardization [19,20]. This class of studies are referred to as *transportability* studies. In addition to standardizing clinical trial results, when applied with rigor, *generalizability* or *transportability* methods can be a valuable tool to estimate treatment effects in the target population using observational studies results. However, few studies have done so [21–23].

Motivated by the use case where we are interested in comparing two existing cancer treatments approved for use in different lines of therapy, we set out to apply *transportability* method to estimate treatment effect in the target population based on results from an observational study. Using a rich observational dataset that contains both per-indication and off-the-label mediation use, our first objective is to compare the efficacy of the two treatments head-to-head using inverse probability of treatment weighting (IPTW) to adjust for confounding in second-line patients. Next, we are interested in using IOPW to adjust for non-representativeness and standardize such results to the population of first-line patients. We will apply PS weighting twice in a row to correct for both nonrandom assignment and non-representativeness of the study sample. We propose this two-step approach as a way of generating RWE especially when few relevant trial results are available. Our study not only explores the opportunity of applying *transportability* methods to observational study results, it also adds to the body of literature where multi-stage weighting was carried out to account for more than one sources of bias (confounding, nonresponse, non-representativeness, etc.) [21,23–25].

Specifically, the two treatments of interest in our study are fulvestrant plus palbociclib and letrozole plus palbociclib. Although they have both been approved for estrogen receptor (ER)-positive, human epidermal growth factor receptor (HER2)-negative metastatic breast cancer (MBC) patients, they were intended for different lines of therapy [26,27]. Fulvestrant has been found to be associated with longer overall survival in women of any menopausal status who had progressed on prior endocrine therapy, when used in combination with palbociclib compared to fulvestrant plus placebo [26]. Letrozole plus palbociclib has been found in another trial to result in longer progression free survival than letrozole alone among postmenopausal women with no prior treatment for their advanced disease [27]. Interestingly, an observational study has found that both treatments have been used as first, second, third and beyond line of therapy [28]. Using RWD from Flatiron Health database, we will first conduct a comparative effectiveness study in the second-line patients with IPTW. The treatment effect obtained will then be standardized to first-line patients using IOPW.

## 2. Materials & methods

### 2.1 Data source

This study used the nationwide Flatiron Health electronic health record (EHR) derived de-identified database. The Flatiron Health database is a longitudinal database, comprising de-identified patient-level structured and unstructured data, curated via technology-enabled abstraction [29,30]. The majority of patients in the database originated from community oncology settings; relative community to academic proportions may vary depending on the study cohort. During the study period, the de-identified data originated from approximately 280 US cancer clinics (~800 sites of care). The data are de-identified and subject to obligations to prevent re-identification and protect patient confidentiality.

Our study used de-identified data from 8,356 ER (+), HER2 (-) MBC patients diagnosed from January 1, 2014, to June 30, 2020, who had at least one line of treatments. Specifically, this general cohort was selected based on the following criteria:

- ICD diagnosis of breast cancer (ICD-9 174.x or 175.x or ICD-10 C50x)
- Evidence of Stage IV or recurrent MBC with a metastatic diagnosis date on after January 1, 2014
- At least 2 documented clinical visits on or after January 1, 2014
- Evidence of treatment with at least one line of therapy for metastatic disease
- Evidence of ER (+) defined as having ER (+) or PR (+) test before or up to 60 days after the start of first-line treatment.
- Evidence of HER2 (-) defined by having a HER2 negative test (IH negative (0–1+), FISH negative/not amplified, or negative NOS) and the absence of a positive test (IHC positive (3+), Fish positive/amplified, positive NOS) before or up to 60 days after of the start date of first-line treatment.
- No greater than 90 days gap between metastasis diagnosis date and first structured activity (vital information, medication administration, a non-cancelled drug order, or a reported laboratory test/result) after MBC diagnosis date (to identify patients who are likely to be missing treatment)

Real world progression was defined as any event with EHR documentation of disease worsening, based on clinicians’ reporting [31].

### 2.2 Comparative effectiveness of fulvestrant-palbociclib vs letrozole-palbociclib in second-line patient cohort

#### 2.2.1 Original (second-line) cohort definition

The cohort of second-line patients were defined based on the following inclusion/exclusion criteria: 1) all female adult patients (at time of second-line therapy initiation) who were treated with fulvestrant-palbociclib or letrozole-palbociclib no later than March 30, 2020 inclusive to allow for at least 90 days of follow-up time 2) excluding patients who were treated with CDK inhibitor (palbociclib, ribociclib, abemaciclib), mTOR inhibitors (everolimus), PI3K inhibitors (alpelisib), and any clinical study drug during first-line as a single drug or in combination therapy. Patients were either treated with fulvestrant-palbociclib or letrozole-palbociclib as a second-line therapy. Fourteen days after the start of second-line therapy was used as the index date for all patients in the cohort since an immediate effect of the treatment on the outcome was not expected. Patients who progressed or died before the index date were removed.

#### 2.2.2 Confounders

The following covariates were extracted from the database: age at initiation of second-line therapy, race, stage at initial breast cancer diagnosis, Eastern Cooperative Oncology Group (ECOG) score within 30 days of second-line therapy start date, time from initial breast cancer diagnosis to MBC diagnosis, and medical practice type. Additionally, the number of metastatic sites recorded, visceral disease (whether metastatic disease is in the lung and/or liver), and bone-only metastasis were also determined before or up to 14 days after the initiation of second-line therapy. These covariates were used as confounders among second-line patients based on data availability and clinical significance. Later in Section 2.3.2, the same covariates were used as effect modifiers for the *transportability* portion of our study.

#### 2.2.3 Endpoint

Real-world progression free survival (rwPFS) was used as our primary outcome in the study [32]. An event was defined as the first documented progression or death, whichever came earlier, that happened no earlier than 14 days after the index date. Date of death was set to be the 15^th^ of each month since death data were only available on a monthly granularity. All patients were censored at the time of last clinical note, or 3 years after the index date, whichever came earlier.

#### 2.2.4 Statistical analysis

**Fig 1:**
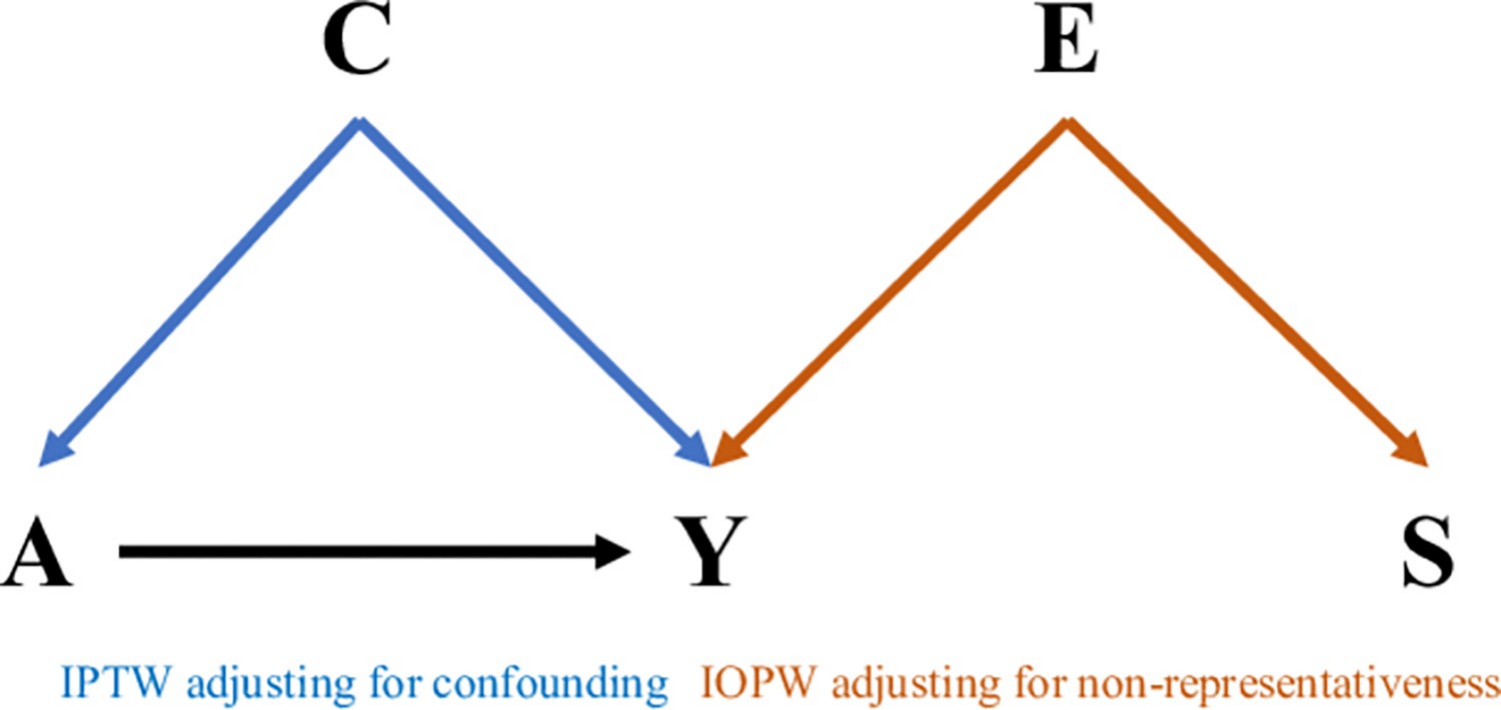
Directed acyclic graph to illustrate our two-step approach to adjust for confounding with inverse probability of weighting (IPTW) and non-representativeness with inverse probability of selection weighting (IOPW). A = treatment assignment; Y = outcome; S = selection into original population from target population; C = confounders; E = treatment effect modifiers.

IPTW was used to adjust for confounding by indication (**Fig 1**). Let ***A*** denote treatment, where *A*_*i*_ = 1 indicates fulvestrant-palbociclib and *A*_*i*_ = 0 letrozole-palbociclib. Let ***C*** denote the vector of confounders. The conditional probability of receiving fulvestrant-palbociclib, ***P***, was estimated using a logistic regression model. Specifically, *P*_*i*_ = *P*(*A*_*i*_ = 1|*C*_*i*_), *i* = 1,2,3…*n*_1_, where *n*_1_ denote the number of patients in the second-line cohort. Stabilized weights (**Table 1**) were estimated for each patient and used to adjust for imbalance of baseline characteristics between two treatment arms [11]. The weights were entered into Cox proportional hazards model with a robust variance estimator for adjustment. Proportional hazards assumption was tested using cox.zph() function in R. Multiple imputation (MI) was used to impute missing data. Specifically, *MI-derPassive* (missing PS variables were imputed before PS was derived) *INT-within* (IPTW was carried out within each imputed datasets) were implemented [33–35].

**Table 1:** Comparison of variables involved in Step 1 and Step 2 analyses.

Cohort		Original (second-line) (*S*_*i*_ = 1)	Target (first-line) (*S*_*i*_ = 0)
Confounder		*C*_*i*_	-
Effect Modifier		-	*E*_*i*_
Treatment		*A*_*i*_	-
Outcome		*Y*_*i*_	-
PS		*P*_*i*_ = *P*(*A*_*i*_ = 1|*C*_*i*_) *i* = 1,2,3…*n*_1_	*Q*_*i*_ = *P*(*S*_*i*_ = 1|*E*_*i*_) *i* = 1,2,3…*n*_1_+*n*_2_
Weights	Step 1	$W_{i}=\frac{A_{i}\times P{(A}_{i}=1)}{P_{i}}+\frac{\left(1-A_{i})\times P(A_{i}=0\right)}{1-P_{i}}$ *i* = 1,2,3…*n*_1_	-
	Step 2	$V_{i}=W_{i}\times \frac{1-Q_{i}}{Q_{i}}\times \frac{P{(S}_{i}=1)}{P(S_{i}=0)}$ *i* = 1,2,3…*n*_1_	0

### 2.3 Transporting findings from second- to the first-line patient cohort

When the treatment effect differs by the levels of other factors, there exists heterogeneity in the causal effect of treatment. We refer to the set of variables as effect modifiers. If at the same time, these effect modifiers also impact the selection of trial participants from the target population of interest, the treatment effect estimated from trial does not represent that in the entire target population [5,14,15]. Post-trial statistical methods can be applied to mitigate this issue [5,15,19,36–38]. Instead of modeling interaction terms directly, trial results can be weighted so the distribution of effect modifiers in the trial resembles that of the target population [5,15,19,36–38]. The weights were created from PS, the conditional probability of trial participation [5,15,19,36–38]. We refer to the scenario where the trial is a subset of the target population as *generalizability*. In contrast, if the trial and target population are disjoint, this is a *transportability* scenario. For example, one trial can be conducted in a patient population when they were diagnosed with a certain condition between 2005–2006, but investigators are interested in *transporting* the trial results to the current patient population who were newly diagnosed with the condition in 2021. We will use IOPW to *transport* the results from Section 2.2 to a first-line patient cohort.

#### 2.3.1 Target (first-line) cohort definition

The first-line cohort was defined using the following inclusion/exclusion criteria: 1) all female adult patients (at time of first-line therapy initiation) who were treated with a first-line MBC therapy no later than March 30, 2020 inclusive to allow for at least 90 days follow-up 2) no evidence of another primary cancer (any occurrence of ICD-9 code 140–208, 209.73, 209.11, 209.29, 209.21) [39] within 3 years before initiation of first-line therapy 3) excluding patients treated with fulvestrant prior to first-line therapy 4) ECOG score 0–2 within 30 days of first-line therapy initiation. No treatment variable was needed for this analysis.

#### 2.3.1 Target (first-line) cohort definition

Similarly defined covariates as in Section 2.2.2 were considered as effect modifiers among the first-line cohort: age at initiation of first-line therapy, race, stage at initial breast cancer diagnosis, ECOG score within 30 days of first-line therapy start date, time from initial breast cancer diagnosis to MBC diagnosis, and medical practice type. Additionally, the number of metastatic sites recorded, visceral disease (whether metastatic disease is in the lung and/or liver), and bone-only metastasis were also determined before or up to 14 days after the initiation of first-line therapy.

#### 2.3.3 Endpoint

Outcome variable was not needed for this analysis.

#### 2.3.4 Statistical analysis

First, the extent to which the distribution of potential effect modifiers differ in the two cohorts was assessed using standardized mean differences (SMD) [11]. Next, data on effect modifiers from the original and target cohorts were concatenated together. An indicator variable denotes whether an individual belongs to the original (*S*_*i*_ = 1) or the target (*S*_*i*_ = 0) cohort. Logistic regression was used to estimate the conditional probability of being in the target cohort, ***Q***, given all effect modifiers ***E***. More formally, *Q*_*i*_ = *P*(*S*_*i*_ = 1|*E*_*i*_), *i* = 1,2,3…*n*_1_+*n*_2_ where *n*_2_ denotes the number of patients in the first-line cohort.

To assess the similarity of the cohorts based on the distribution of ***Q***, Tipton index was computed [38]. Tipton index was defined as $\int \sqrt{f(p_{1})f(p_{2})}dp$, where *p*_1_ and *p*_2_ denote vectors of propensity scores estimated in the original and target cohorts respectively [38]. Tipton index is unit-less similarity metric and ranges from 0 to 1. A higher level of Tipton index indicates two populations are highly similar (above 0.8) [38]. That means the application of *transportability* methods will involve less extrapolation, and thus invoke higher confidence of the results [38].

Next, IOPW was implemented to adjust for non-representative of the original cohort as compared to the target population of first-line patients (**Fig 1**). The weights for adjusting for differential distribution of effect modifiers were estimated as below, where the weights from Section 2.2.4 were multiplied by the odds of being in the original cohort [19]. To *transport* the treatment effect from Section 2.2 to the new target population, Cox proportional hazards model with a robust variance estimator was applied to individuals in the original cohorts *only*, adjusting for weights ***V*** that was estimated using two cohorts combined, including no covariates in the model **(Table 1)**. Note that neither treatment nor outcome variable from the new target population was used in the analysis. Only potential effect modifiers were used to estimate the weights for the Cox model. Similarly to the IPTW analysis described in Section 2.2.4, *MI-derPassive INT-within* was used to impute missing data [33,34]. Specifically, covariates from both original and target cohorts, treatment and outcome variables from the original cohort were included in the imputation model [35]. Last, SMD was again used to quantify the difference of two populations after weighting. All statistical analyses were conducted in R version 4.0.3 [40]. R code used for modeling can be found at https://github.com/yling2019/SERD_transportability.

## 3. Results

**Fig 2:**
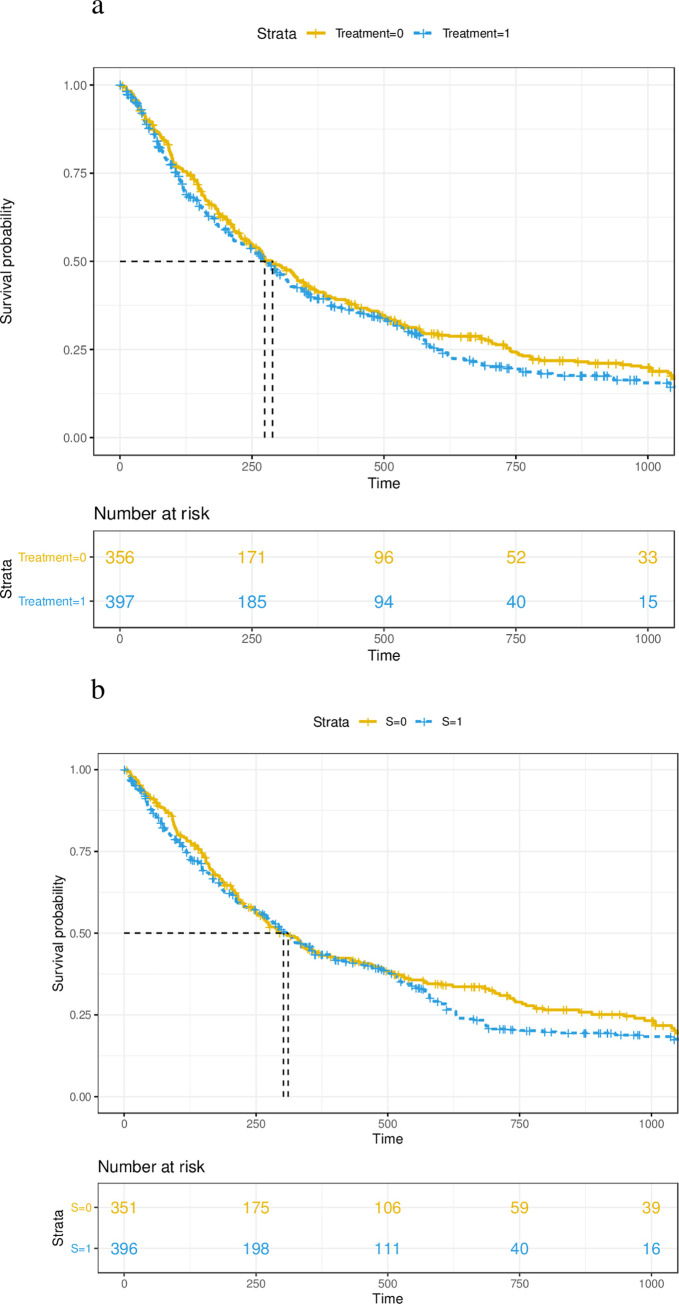
Kaplan Meier Curves in a) IPTW adjusted (for confounding only) cohort of second-line patients b) IPTW and IOPW adjusted (for both confounding and non-representativeness) cohort of first-line patients. Time is shown in days. Note that sample size was impacted by weighting in our analyses.

There were 752 patients in the original second-line cohort, 397 of which were treated with fulvestrant-palbociclib and 355 with letrozole-palbociclib (Table 2). Compared to patients in letrozole-palbociclib arm, patients treated with fulvestrant-palbociclib were older with a larger proportion of White patients, and ECOG score of 0, who were initially diagnosed with Stage 0–3 (Table 2). The gap between initial breast cancer diagnosis and MBC diagnosis tended to be more than 1 years for patients treated with fulvestrant-palbociclib. The median follow-up time for fulvestrant-palbociclib and letrozole-palbociclib arms were 212.0 days (mean = 312.6, standard deviation (SD) = 288.0) and 260.0 days (mean = 382.6, SD = 335.2) respectively. There were a total of 528 events observed, 280 of which were in fulvestrant-palbociclib arm and 248 in letrozole-palbociclib arm. After IPTW, SMD for all covariates were below 0.1 for all m = 36 imputed datasets. For each imputed dataset, proportional hazards assumptions were met by testing Schoenfeld residuals (median p-value 0.9517, min 0.7955, max 0.9974). The IPTW adjusted hazard ratio (HR) was 1.11 with 95% confidence interval [0.93, 1.32]. This is consistent with observations from the Kaplan-Meier curve (**Fig 2A**), where the two curves are very close to each other, even though the probability of survival was slightly higher in the letrozole-palbociclib arm.

**Table 2:** Baseline characteristics before and after inverse probability of treatment weighting (IPTW) among the second-line population.

	Before IPTW			After IPTW		
	Letrozobe + Palbociclib (N = 355)	Fulvestrat + Palbociclib (N = 397)	SMD	Letrozobe + Palbociclib (N = 356)	Fulvestrat + Palbociclib (N = 397)	SMD (median (range))
Age (mean (SD))	65.45 (11.27)	67.64 (10.70)	0.200	66.80 (11.07)	66.74 (11.10)	0.006 (0,0.014)
Race (%)			0.133			0.017 (0.008,0.029)
White	252 (75.7)	296 (77.9)		270.4 (76.0)	300.7 (75.7)	
Black or African American	30 (9.0)	27 (7.1)		27.6 (7.7)	31.7 (8.0)	
Asian	13 (3.9)	8 (2.1)		11.5 (3.2)	13.9 (3.5)	
Other	38 (11.4)	49 (12.9)		46.2 (13.0)	50.6 (12.8)	
Missing	22 (6.2)	17 (4.3)				
Stage (%)			0.555			0.014(0.008,0.021)
I	48 (14.5)	58 (15.8)		54.8 (15.4)	61.8 (15.6)	
II	89 (26.8)	156 (42.5)		127.4 (35.8)	141.3 (35.6)	
III	63 (19.0)	93 (25.3)		80.6 (22.7)	88.5 (22.3)	
IV	132 (39.8)	60 (16.3)		92.9 (26.1)	105.4 (26.5)	
Missing	23 (6.5)	30 (7.6)				
ECOG (%)			0.194			0.011 (0.002,0.03)
0	92 (36.2)	126 (45.0)		142.6 (40.1)	158.3 (39.9)	
1	112 (44.1)	113 (40.4)		153.0 (43.0)	171.1 (43.1)	
2	38 (15.0)	31 (11.1)		42.5 (11.9)	48.0 (12.1)	
> = 3	12 (4.7)	10 (3.6)		17.6 (5.0)	19.6 (4.9)	
Missing	101 (28.5)	117 (29.5)				
Number of metastatic sites (%)			0.058			0.007 (0.002,0.012)
1	158 (44.6)	173 (43.7)		153.8 (43.2)	171.9 (43.3)	
2	103 (29.1)	109 (27.5)		99.6 (28.0)	111.5 (28.1)	
> = 3	93 (26.3)	114 (28.8)		102.3 (28.8)	113.6 (28.6)	
Missing	1 (0.3)	1(0.3)				
Visceral Disease = 1 (%)	141 (39.7)	178 (44.8)	0.104	156.5 (44.0)	175.6 (44.2)	0.004 (0,0.011)
Bone Only Disease = 1 (%)	121 (34.1)	124 (31.2)	0.061	113.7 (32.0)	126.7 (31.9)	0.004 (0,0.014)
Time from initial breast cancer to MBC diagnosis (%)			0.556			0.006 (0.001,0.01)
< = 1 year	149 (42.0)	70 (17.7)		103.8 (29.2)	116.1 (29.3)	
1–5 years	78 (22.0)	137 (34.6)		100.9 (28.4)	112.6 (28.4)	
>5 years	128 (36.1)	189 (47.7)		151.0 (42.5)	168.3 (42.4)	
Missing	0(0)	1(0.3)				
Practice Type = COMMUNITY (%)	326 (91.8)	362 (91.2)	0.023	323.6 (91.0)	362.3 (91.3)	0.013 (0.003,0.023)

SMD: Standardized mean difference. Note that sample size was impacted by weighting in our analyses.

The target population consisted of 3,109 patients who were eligible to receive first-line treatment of their MBC (**Table 3**). Compared to the original second-line cohort, this target first-line patient population was younger (**Table 3**). They also had a larger Black representation, more people diagnosed with *de novo* MBC, less metastatic sites, and a smaller gap between initial breast cancer diagnosis and MBC diagnosis (**Table 3**). Mean Tipton index among all m = 25 multiply imputed datasets was 0.81 (SD = 0.01) before weighting, indicating that the two populations are similar enough to carry out a *transportability* study [16]. After IOPW, SMD for the covariates were below 0.1 for all imputed datasets. Even though SMD of ECOG scores for some imputed datasets were above 0.1, the mean SMD was 0.10 with SD 0.01. For each imputed dataset, proportional hazards assumptions were met by testing Schoenfeld residuals (median p-value 0.8947, min 0.4424, max 0.9988). The IOPW adjusted hazard ratio was 1.12 with 95% confidence interval [0.90, 1.39]. This result is also consistent with Kaplan-Meier curve (**Fig 2B**), where both median survival times and the overall curves are very similar to each other.

**Table 3:** Baseline characteristics before and after inverse probability of selection weighting (IOPW).

	Before IOPW			After IOPW		
	First-line population (N = 3,109)	Second-line population (N = 752)	SMD	First-line population (N = 3.109)	Second-line population (N = 746)	SMD (median (range))
Age (mean (SD))	64.86 (12.61)	66.61 (11.02)	0.147	64.86 (12.61)	64.98 (11.48)	0.01 (0,0.017)
Race (%)			0.122			0.061 (0.02,0.084)
White	2066 (73.5)	548 (76.9)		2280.0 (73.3)	549.2 (73.6)	
Black or African American	324 (11.5)	57 (8.0)		361.0 (11.6)	79.5 (10.7)	
Asian	73 (2.6)	21 (2.9)		84.0 (2.7)	25.6 (3.4)	
Other	349 (12.4)	87 (12.2)		384.0 (12.4)	92.1 (12.3)	
Missing	297 (9.6)	39 (5.2)				
Stage (%)			0.229			0.034 (0.019,0.052)
I	394 (13.8)	106 (15.2)		454.0 (14.6)	107.5 (14.4)	
II	854 (29.9)	245 (35.1)		973.0 (31.3)	232.5 (31.1)	
III	523 (18.3)	156 (22.3)		584.0 (18.8)	132.1 (17.7)	
IV	1089 (38.1)	192 (27.5)		1098.0 (35.3)	274.4 (36.8)	
Missing	249 (8.1)	53 (7.0)				
ECOG (%)			0.334			0.103 (0.09,0.117)
0	1543 (49.6)	218 (40.8)		1543.0 (49.6)	349.9 (46.9)	
1	1131 (36.4)	225 (42.1)		1131.0 (36.4)	282.1 (37.8)	
2	435 (14.0)	69 (12.9)		435.0 (14.0)	111.2 (14.9)	
> = 3	0 (0.0)	22 (4.1)		0.0 (0.0)	3.2 (0.4)	
Missing		218 (29.0)				
Number of Metastatic Sites (%)			0.171			0.022 (0.013,0.032)
1	1508 (52.3)	331 (44.1)		1604.0 (51.6)	380.8 (51.0)	
2	741 (25.7)	212 (28.3)		836.0 (26.9)	196.6 (26.3)	
> = 3	634 (22.0)	207 (27.6)		669.0 (21.5)	169.0 (22.6)	
Missing	226 (7.3)	2 (0.3)				
Visceral Disease = 1 (%)	1196 (38.5)	19 (42.4)	0.081	1196.0 (38.5)	290.6 (38.9)	0.007 (0.003,0.024)
Bone Only Disease = 1 (%)	967 (31.1)	245 (32.6)	0.032	967.0 (31.1)	234.9 (31.5)	0.007 (0,0.014)
Time from initial breast cancer to MBC diagnosis (%)			0.236			0.042 (0.034,0.049)
< = 1 year	1253 (40.3)	219 (29.2)		1253.0 (40.3)	306.0 (41.0)	
1–5 years	748 (24.1)	215 (28.6)		748.0 (24.1)	167.0 (22.4)	
> 5 years	1107 (35.6)	317 (42.2)		1108.0 (35.6)	273.4 (36.6)	
Missing	1 (0.03)	1 (0.1)				
Practice Type = COMMUNITY (%)	3090 (99.4)	688 (91.5)	0.386	3090.0 (99.4)	739.7 (99.1)	0.034 (0.031,0.038)

SMD: Standardized mean difference. Note that sample size was impacted by weighting in our analyses.

## 4. Discussion

Initially using IPTW to address confounding, we have estimated the HR of fulvestrant-palbociclib compared to letrozole-palbociclib in second-line patients to be 1.11 with 95% confidence interval [0.93, 1.32]. Next, we have additionally applied IOPW to *transport* these results to a first-line patient population and obtained a HR of 1.12 with 95% confidence interval [0.90, 1.39]. For both patient cohorts, the small beneficial treatment effect of letrozole-palbociclib was not statistically significant. The similarity of the two hazard ratios indicates little treatment effect modification from selected demographic and clinical variables in our study.

Our study was aimed to generate RWE using the rich dataset from Flatiron Health. However, we are aware of the level of such evidence, given our results were *transported* from a cohort study. Thus, our results need to be considered in conjunction with other types of evidence in the literature. One future direction is to conduct an observational study directly in first-line patient cohort, comparing the two treatments of interest using off-the-label medication use. There has been only one clinical trial comparing fulvestrant-palbociclib vs letrozole-palbociclib as a first-line therapy [41]. In the trial, 486 MBC women with no prior treatment for their metastatic disease were randomly assigned with 1:1 ratio to either treatment arms with a primary end point investigator-assessed progression free survival (PFS). The HR was 1.13 with 95% 0.89–1.45 and similar result was also observed when stratified by type of metastatic disease and visceral involvement. Although there are differences between this trial and our study (e.g. study design, study end point), neither found fulvestrant-palbociclib to have a different treatment effect from letrozole-palbociclib.

There are many limitations to our study. First and foremost, *transportability* studies usually assume the outcome generating functions of the two populations to be the same. In our real-world example, this means that we assumed progression or death happened through a similar pathway in the second- and first-line cohorts after treatment with fulvestrant-palbociclib relative to treatment with letrozole-palbociclib. While impossible to test, we can assess the plausibility of this assumption based on biological and epidemiological considerations. For example, consider the fact that all second-line patients have already been exposed to a first-line treatment. This drug exposure history may affect the outcome directly, or more importantly have a synergistic effect with the treatment of interest that alters its effect. If this were the case, the treatment effect would be impossible to estimate simply because we do not know how first-line therapy patients will be treated in the future, nor do we have the actual data to adjust for prior history of treatment. In addition, treatments in neoadjuvant or adjuvant setting could also be part of a patient’s drug exposure history, which was not captured in our study. Thus, we must be willing to assume that the effect of such treatments would have a negligible impact on the treatment effect. Additionally, while our data example illustrated a *transportability* scenario (where trial sample is disjoint from target population) [19], our original and target populations are not independent from each other. All patients in the second-line cohort were also eligible to be included in the first-line cohort, although the covariate space differs depending on the respective cohort they are in. While these two datasets were related, we believe that the concept of *transportability* is still appropriate here. This is because we believe our first-line cohort is still more representative of a typical first-line cohort in the real-world. We also acknowledge that the validity of our findings in the first-line cohort is dependent on the internal validity of results in the first-line cohort, which isn’t as strong as a clinical trial. However, no such trial has been conducted and therefore we have conducted this study to generate RWE. Lastly, we are limited by our dataset and may not capture all potential confounder and effect modifiers in our analyses.

We have demonstrated the feasibility of *transporting* results from a cohort study as a way to generate RWE when limited clinical evidence is available. Investigators need to exercise caution in interpretation of the findings that result from such applications. Specifically, assumptions underlying validity of such methods needs to be met and the challenges of achieving this may be higher when *transporting* from observational findings than randomized clinical trials. As more well designed observational studies have been conducted to emulate clinical trials [13,42,43], such application will further motivate methodologists and applied researchers to conduct more high-quality observational studies and make better use of such studies to provide RWE [44].
